# Role of Wildlife in Emergence of Ebola Virus in Kaigbono (Likati), Democratic Republic of the Congo, 2017

**DOI:** 10.3201/eid2609.191552

**Published:** 2020-09

**Authors:** Sophie Gryseels, Placide Mbala-Kingebeni, Innocent Akonda, Roger Angoyo, Ahidjo Ayouba, Pascal Baelo, Daniel Bamuleka Mukadi, Elie Bugentho, Trenton Bushmaker, Christelle Butel, Sébastien Calvignac-Spencer, Eric Delaporte, Birgit De Smet, Ariane Düx, François Edidi-Atani, Robert Fischer, Corneille Kahandi, Jimmy Kapetshi, Servet Kimbonza Sumba, Léonce Kouadio, André Malekani Bendeke, Claude Mande, Guy Midingi Sepolo, Joseph Moudindo, Eitel Mpoudi Ngole, Prescott Musaba, Patrick Mutombo, Innocent Ndong Bass, Casimir Nebesse, Steve Ngoy, Simon-Pierre Ndimbo Kumogo, Stephanie N. Seifert, Jacques Tanzito, Dudu Akaibe, Nicaise Amundala, Kevin K. Ariën, Guy-Crispin Gembu, Fabian H. Leendertz, Herwig Leirs, Jean-Claude Mukinzi, Vincent Munster, Jean-Jacques Muyembe-Tamfum, Martine Peeters, Erik Verheyen, Steve Ahuka-Mundeke

**Affiliations:** University of Arizona, Tucson, Arizona, USA (S. Gryseels); KU Leuven, Leuven, Belgium (S. Gryseels);; University of Antwerp, Antwerp, Belgium (S. Gryseels, H. Leirs, E. Verheyen);; Université de Montpellier, Montpellier, France (P. Mbala-Kingebeni, A. Ayouba, C. Butel, E. Delaporte, J. Moudindo, E. Mpoudi Ngole, I. Ndong Bass, M. Peeters);; Institut National de Recherche Biomédicale, Kinshasa, Democratic Republic of the Congo (P. Mbala-Kingebeni, F. Edidi-Atani, J. Kapetshi, S. Kimbonza Sumba, G. Midingi Sepolo, D. Bamuleka Mukadi, S.P. Ndimbo Kumogo, J.J. Muyembe-Tamfum, S. Ahuka-Mundeke);; Division Provinciale de la Sante, Buta (Bas-Uele), Democratic Republic of the Congo (I. Akonda);; Programme National de Lutte Contre le Sida, Buta (I. Akonda);; Centre de Surveillance de la Biodiversité, Kisangani, Democratice Republic of the Congo (R. Angoyo, P. Baelo, E. Bugentho, C. Kahandi, A. Malekani Bendeke, C. Mande, P. Musaba, P. Mutombo, C. Nebesse, S. Ngoy, J. Tanzito, D. Akaibe, N. Amundala, G.C. Gembu, J.C. Mukinzi);; National Institutes of Health, Hamilton, Montana, USA (T. Bushmaker, R. Fischer, S.N. Seifert, V. Munster);; Robert Koch Institute, Berlin, Germany (S. Calvignac-Spencer, A. Düx, L. Kouadio, D. Bamuleka Mukadi, F.H. Leendertz);; Institute of Tropical Medicine, Antwerp (B. De Smet, K.K. Ariën);; Laboratoire Central de la Pathologie Animale, Bingerville, Côte d’Ivoire (L. Kouadio);; University of Kisangani, Kisangani (C. Mande, P. Musaba, C. Nebesse, D. Akaibe, N. Amundala, G.C. Gembu, J.C. Mukinzi);; Centre de Recherches sur les Maladies Émergentes, Re-émergentes et la Médecine Nucléaire, Yaoundé, Cameroon (J. Moudindo, E. Mpoudi Ngole, I. Ndong Bass);; Royal Belgian Institute of Natural Sciences, Brussels, Belgium (E. Verheyen)

**Keywords:** Ebola virus, ecology, infectious disease reservoir, mammals, zoonoses, viruses, Ebola virus infection, viral zoonoses, Democratic Republic of the Congo, Ebola virus disease, Potamochoerus porcus, Eidolon helvum

## Abstract

After the 2017 Ebola virus (EBOV) outbreak in Likati, a district in northern Democratic Republic of the Congo, we sampled small mammals from the location where the primary case-patient presumably acquired the infection. None tested positive for EBOV RNA or antibodies against EBOV, highlighting the ongoing challenge in detecting animal reservoirs for EBOV.

The animal reservoir(s) for Ebola virus (EBOV) remain unclear. Although substantial evidence suggests several bat species can host EBOV and other filoviruses (*1*–*8*), it cannot be ruled out that other, less frequently surveyed mammal groups could also host these viruses or play a role in their ecology (*9*). An EBOV outbreak in humans implies that EBOV had been circulating among wildlife where the primary case-patient contracted the infection. If the primary case-patient and his or her activities before becoming ill are known, this information provides an opportunity for EBOV wildlife surveillance closely focused in space and season.

In late March 2017, signs and symptoms of hemorrhagic fever developed in an inhabitant of Kaigbono, a village in Likati district in northern Democratic Republic of the Congo (Figure 1, panel A) (*10*). In subsequent weeks, 2 probable and 5 confirmed cases of Ebola virus disease followed in nearby villages (Figure 1, panel B) (*10*,*11*). The World Health Organization officially declared this outbreak over on July 2, 2017. Our team arrived in Kaigbono on July 5, 2017, to investigate potential EBOV circulation among local wildlife.

**Figure 1:**
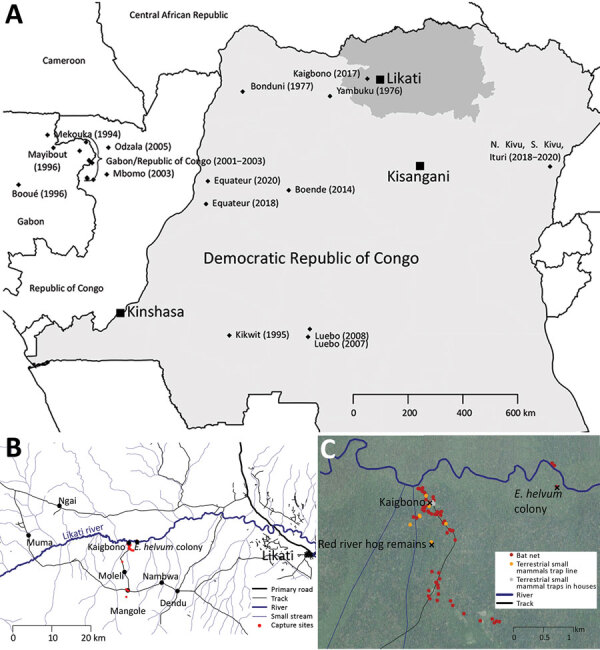
Locations of human Ebola virus (EBOV) outbreaks in Central Africa and capture site of potential wildlife reservoirs in study of role of wildlife in emergence of Ebola virus, Democratic Republic of the Congo, 2017. A) Reported human EBOV outbreaks in central Africa. Diamonds indicate the approximate locations where each outbreak started. Outbreak year(s) are shown in brackets. Bas-Uele province is highlighted in dark gray. B) Overview of the area where the 2017 EBOV outbreak occurred (Likati Health Zone, Bas-Uele, Democratic Republic of the Congo). Black dots indicate villages and *Eidolon helvum* bat colony; red dots indicate sites where mammals were captured in this study. C) Study area at and around Kaigbono village, with most capture sites indicated.

## The Study

The primary case-patient spent the weeks preceding his illness in and around his home village of Kaigbono, a settlement of <50 inhabitants near the Likati River (Figure 1). The village is accessible only via the Likati River or narrow forest paths. The primary case-patient often collected fish from fishermen along the river and transported it to the village. 

This primary case-patient ate cooked meat from a red river hog (*Potamochoerus porcus*) »13 days before symptom onset. Other persons had found the dead hog in the forest ≈700 m from the village. Up to 4 Kaigbono villagers (none of whom became ill) collected the meat around the upward shoulder area of the hog, reportedly leaving the rest of the carcass because the meat touching the ground and the internal organs had already decomposed. Two of these persons prepared and cooked the meat, which subsequently was shared by ≈10–20 persons in Kaigbono, including the primary case-patient (the only person in whom febrile illness developed). On July 10 we retrieved a skull of a red river hog at the site described as the location where the abovementioned hog was found (Figure 1, panel C). Another potential zoonotic exposure occurred »7 days before symptom onset in the primary case-patient when he brought home a large bat. Other persons had hunted and killed the bat, probably at the site of a large seasonal colony of straw-colored fruit bats at the Likati River (*Eidolon helvum*; Figure 1, panels B, C). Villagers reported that the colony arrives annually in March and leaves in July; we observed that most of the *E. helvum* bats left the site during July 16–19. Given the colony’s large size (at least several thousand), most bats hunted during this season probably belong to *E. helvum*. However, we observed that *Hypsignathus monstrosus* and *Epomops franqueti*, other bat species in which EBOV RNA has been documented (*3*), also might have been occasionally hunted. The primary case-patient’s wife prepared (removed its internal organs) and grilled the bat. Only the primary case-patient ate the bat. His wife did not report a fever. 

From July 6, 2017 through August 18, 2017, we trapped 476 small mammals (rodents, shrews, and bats) and acquired samples from 11 mammals hunted by local inhabitants. None of the animals showed signs of illness. Of these animals, we euthanized and collected organ samples (preserved in RNALater) of 388 (when possible, we also collected dried blood spots and oral, urogenital, and/or rectal swab specimens from these animals); collected only dried blood spots and oral, urogenital, and/or rectal swab specimens of 79; and did not sample 20. We collected bat fecal samples from plastic sheets fixed to trees underneath the *E. helvum* bat colony (Figure 1, panels B, C). We also swabbed the exterior of the skull of the abovementioned red river hog and extracted its molars.

**Figure 2:**
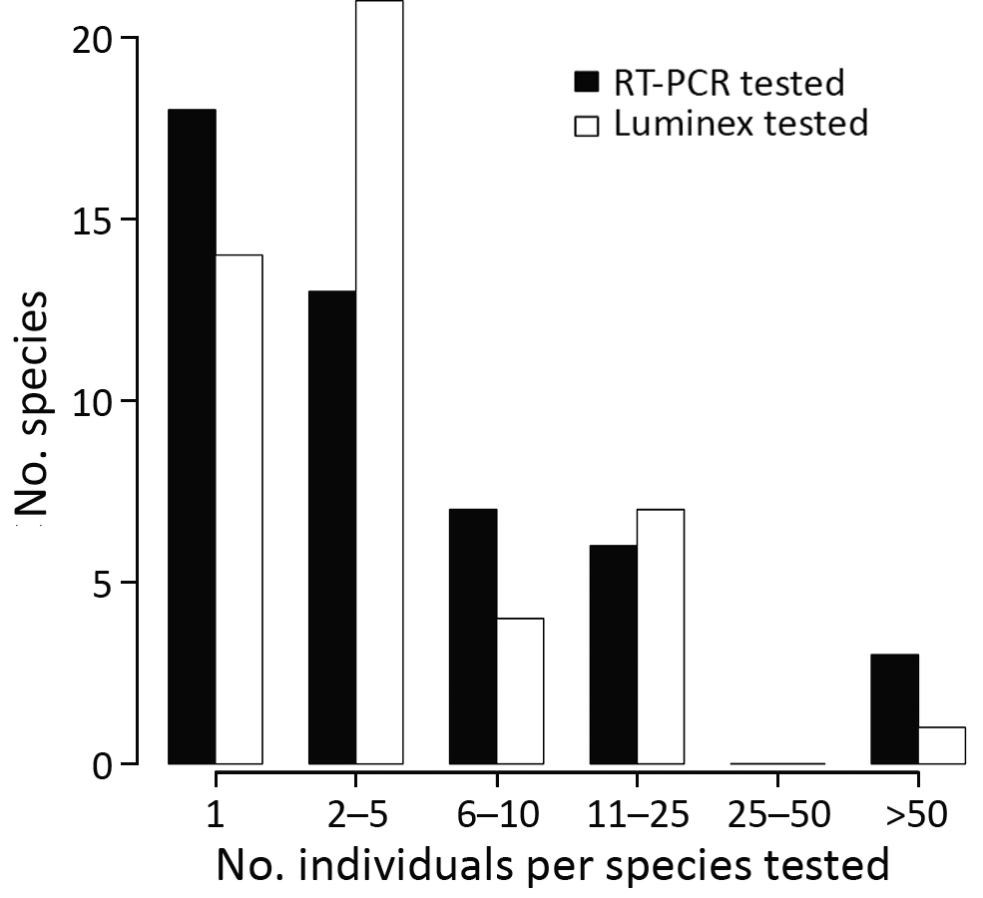
Abundance distribution of mammal species tested for Ebola virus and Sudan virus RNA using quantitative RT-PCR and for antibodies against ebolaviruses using the Luminex assay (Luminex Corporation, https://www.luminexcorp.com), for the set of specimens sampled in and around Kaigbono (Likati Health Zone, the Democratic Republic of the Congo) in 2017 that were determined to the species level. Each successfully tested environmental fecal sample is assumed to represent a single *Eidolon helvum* bat specimen (full descriptions available in Appendix Table). RT-PCR, reverse transcription PCR.

We extracted RNA from >1 organ, blood, and/or fecal samples of 419 of the 467 sampled animals and from samples swabbed from the skull and the molar pulp remains of the red river hog. We performed multiplex quantitative reverse transcription PCR (qRT-PCR) targeting EBOV and Sudan virus L gene, as previously described (*12*). We further tested RNA extract from 91 fecal samples and 1 urine sample collected at the *E. helvum* bat colony, although 47 of these samples showed signs of PCR inhibition. None of the samples of the total of 465 individual animals or environmental feces were qRT-PCR positive (Figure 2; Appendix Table).

We used a 10-antigen Luminex assay (Luminex Corporation, https://www.luminexcorp.com) to test dried blood spots of 272 animals and 92 fecal samples for antibodies against EBOV, as previously described (*2*,*13*). None of these samples could be considered positive for antibodies against EBOV (Appendix Figure).

Cytochrome b, 16S, or 12S gene PCR was attempted and Sanger sequenced on a subset (n = 334) of specimens for host species confirmation. We deposited sequences in GenBank under accession nos. MN597466–MN597893.

We distinguished 47 different mammal species (4 could not be assigned to a known species catalogued in GenBank) from 34 different genera across 359 specimens. For 268 of these, genetic information was necessary to identify the species, as species identification was not possible or not done correctly in the field (Appendix Table). Most species had low sample sizes with little power to detect low virus prevalences (Figure 2; Appendix). For an additional 67 nongenotyped animals, the genus could be unambiguously determined based on field morphology. We did not determine a genus or species for 62 animals.

## Conclusions

Before his illness, the primary case-patient of the 2017 EBOV outbreak in Likati had eaten prepared meat from a red river hog and a fruit bat, probably *E. helvum*. He had contact with the uncooked carcass of the bat but not of the hog. The meat of the bat and the hog were prepared by others who had not fallen ill but whose serologic status is unknown. The hog had been dead for several days before butchering and cooking, causing us to question the infectiousness of any virus present in the meat. The susceptibility of the bat species *E. helvum* to EBOV is also questionable because experimental data suggests EBOV could be refractory in *E. helvum* cells (*14*). Thus, we can neither exclude nor confirm which or if either of these animals sparked the Likati 2017 EBOV outbreak.

We started collecting wildlife specimens ≈3 months after the onset of the human outbreak, a time lag potentially important to local natural transmission dynamics yet still within similar seasonal conditions. We ceased sampling 6 weeks later when seasonal changes occurred (e.g., emigration of the *E. helvum* bat colony). Despite this directed sampling, we did not find evidence for EBOV RNA (n = 465 animals tested) or antibodies against EBOV (n = 364) in any wildlife specimen. Because we used a qRT-PCR specific to EBOV and Sudan virus, we cannot exclude the presence of RNA of other filoviruses in the samples. However, the Luminex assay would have revealed any antibodies against related filoviruses.

As noted in previous surveillance studies following an EBOV outbreak (*15**;* reference *1* in Appendix), the high mammal species diversity in the Congo basin rainforest, combined with the remoteness of the outbreak site, complicates the collection of a sufficient sample size for all potentially relevant taxa (Figure 2; Appendix). Furthermore, many small mammals are difficult to identify at a species or genus level on the basis of morphology alone (Appendix). Therefore, we emphasize that EBOV and other virus surveillance in small mammals requires molecular identification of the host species. 

Because all human EBOV outbreaks start from a spillover event from wildlife, knowledge on exactly which wildlife species are involved in EBOV natural ecology would provide a more precise geographical and seasonal risk map for human EBOV disease outbreaks. Therefore, despite the challenges highlighted by this study, investing in increased surveillance of African forest wildlife to find EBOV reservoirs could greatly benefit public health preparedness for the devastating disease caused by this virus. 

Appendix Additional materials, methods, results, and discussions about serological testing, mammal species identifications, EBOV detection power, and the source of EBOV infection of the primary case-patient.
