# Supplementary material for: Role of Wildlife in Emergence of Ebola Virus in Kaigbono (Likati), Democratic Republic of the Congo, 2017
Source: Emerg Infect Dis. 2020 Sep;26(9):2205–9. doi: 10.3201/eid2609.191552 (PMC7454093; doi:10.3201/eid2609.191552)
Supplement: Appendix — Additional materials, methods, results, and discussions about serological testing, mammal species identifications, EBOV detection power, and the source of EBOV infection of the primary case-patient. [file 19-1552-Techapp-s1.pdf]

Article DOI: https://doi.org/10.3201/eid2609.191552

Role of Wildlife in Emergence of Ebola Virus, Kaigbono (Likati), Democratic Republic of the Congo, 2017

Appendix

Detailed Methods

Wildlife Capture and Sampling

Terrestrial small mammals were captured using Sherman LFA live traps (Sherman Live Trap Co., Tallahassee, FL, USA), snap traps, and pitfall traps of which 10–35 each were placed intermittently 5m apart along 10 different lines in primary and secondary forest habitat (Figure 1, main text) or placed within 10 habitations in Kaigbono village (Sherman live traps only). Traps were checked every morning and (re-)baited in the late afternoon with palm nuts. A total of 5460 trap nights (number of traps × nights set) were reached. Bats were captured in mist nets placed at 72 different sites over a total of 237 netting nights (Figure 1, main text). A traditional squirrel trap was placed in trees for 20 nights. When available, wildlife hunted by local inhabitants were sampled as well.

To collect a larger number of bat feces samples, plastic sheets were fixed to trees underneath a large straw-colored fruit bat (*Eidolon helvum*) colony (location indicated in Figure 1, main text). Initially, one and then subsequently two sheets were left out for two nights, and we pooled all the collected feces into one tube with RNAlater per session and collected pooled urine on Serobuvard filter paper. Afterwards, respectively three, eight, and six sheets were left out for one night across three consecutive nights, and feces were collected individually from these sheets. A total of 96 fecal samples and one urine sample were collected. We observed that most of the *E. helvum* bats had left the site between July 16th and 19th 2017, after which we ceased feces collections.

Most animals captured alive in Sherman live traps, pitfall traps and mist nets were euthanized via isoflurane inhalation. We captured a total of 476 individual animals and a further

11 were acquired via the local community. For 388 captured individual animals we performed full sampling: liver, lung, spleen and kidney specimens were collected in RNAlater, and if possible whole blood was collected on Serobuvard (LDA 22, Zoopole, France) or Whatmann filter paper (N = 245), henceforth called dried blood spots (DBS). For 55 specimens also 10–70µL of whole blood was stored in 310µL AVL (Qiagen) and the remainder set to settle overnight, after which serum and plasma were extracted and stored separately at ambient temperature for 7–10 days, after which they were stored at -20°C. Oral and rectal swabs (stored in RNAlater) were also collected for 275 of these fully sampled animals, and for 59 an additional urogenital swab. For 65 of the captured individual animals, only whole blood, an oral swab and a rectal swab (stored in RNAlater) were collected after which the animals were released. From 14 animals, only oral and rectal swabs were collected, after which the live animal was released or the carcass returned to the owner. Twenty animals were not sampled but carcasses were collected. All carcasses were stored in 10% formalin.

We furthermore retrieved the skull of an ≈4-month-old red river hog carcass that the primary case-patient of this outbreak had been exposed to. We swabbed the exterior of this skull (swabs stored in RNAlater) and extracted molars, which were stored at room temperature and later at -20°C.

Field work was carried out under a permanent research permit from the Institut Congolais pour la Conservation de la Nature (ICCN) and the Congolese Ministry of Environment granted to the Centre de Surveillance de la Biodiversité of the University of Kisangani. All work with live animals was furthermore approved by the Institutional Animal Care and Use Committee of the Rocky Mountain Laboratories (RML) under permit number 2015–010. Animal work was conducted adhering to RML’s guidelines for animal use, and followed the guidelines and basic principles in the United States Public Health Service Policy on Humane Care and Use of Laboratory Animals, and the Guide for the Care and Use of Laboratory Animals by certified staff in an Association for Assessment and Accreditation of Laboratory Animal Care (AAALAC) International accredited facility.

All tissue specimens are stored at the Institut National de Recherche Biomédicale (INRB) in Kinshasa, DRC, and all animal carcasses in formalin are stored at the Centre de surveillance de la Biodiversité (CSB) of the University of Kisangani, Kisangani, DRC.

Molecular Detection of EBOV

After the first 2 weeks of field work, the first batch of samples were taken to INRB, Kinshasa, for initial testing. A first set of whole blood (fresh or in AVL) and/or DBS of 45 representative animals and one fecal sample supernatant were tested for EBOV and SUDV using the Idylla platform (*2*). In brief, 50–200µL of whole blood and 100–200µL AVL or 2–7 DBS and 400µL AVL were loaded onto the Idylla cartridges, which each contain a chamber with automatic RNA extraction, and four chambers in which RT-PCR of both an internal positive control (Phocine morbillivirus RNA) and EBOV target takes place, and a fifth RT-PCR chamber for SUDV detection (*2*). For three specimens the internal positive control was not amplified, also not in test repeats. Due to the low-throughput of the Idylla system and relatively low volume of the blood samples relative to the organ samples, we further continued with EBOV testing using a qRT-PCR assay on RNA extracted from organ samples.

RNA extraction of organ samples (lung, liver, spleen) was carried out using the Qiagen RNeasy extraction kit. Approximately 30 mg of organ tissue in RLT was homogenized with a single-use 5-mm stainless steel bead using Qiagen TissueLyser on 25 Hz for 5 minutes. The extraction protocol was continued according to manufacturer's instructions but leaving out the DNase treatment step. Swab samples and whole blood stored in AVL were extracted with Qiagen QIAamp Viral RNA Mini Kit. Total nucleic acids in DBS and fecal samples were extracted with Biomerieux Nuclisens Minimag kit. We drilled through a molar tooth removed from the red river hog skull and scraped the inside of this drill hole where the remains of the molar pulp would be. RNA was extracted from this material with Qiagen RNeasy extraction kit according to manufacturer's instructions but leaving out the DNase treatment step.

All extracted samples were tested with a multiplex qRT-PCR targeting EBOV and SUDV L gene on a Mic qPCR Cycler (Bio Molecular Systems), as described in de Wit et al., 2016 (*3*). EBOV primers and probes are: D1–1F_EBOV_L CAGCCAGCAATTTCTTCCAT, D1–1R_EBOV_L TTTTCGGTTGCTGTTTCTGTG, D1–1P1_EBOV_L FAM-ATCATTGGC/ZEN/RTACTGGAGGAGCAG-3IABkFQ and D1–1P2_EBOV_L FAM-TCATTGGCG/ZEN/TACTGGAGGAGCAGG-3IABkFQ. SUDV primers and probe are: D1–4F_SUDV_L CAGAAGACAATGCAGCCAGA, D1–4R_SUDV_L TTGAGGAATATCCCACAGGC, D1–4P_SUDV_L HEX-CTGCTAGCTTGGCCAAAGTCACAAG-ZEN-IABkFQ.

Using the multiplex qRT-PCR described above, either all three organ tissue types were tested per individual animal (N = 207), two (N = 163) or one (N = 15). For 220 of the 385 organ-tested animals also a blood sample (DBS or whole blood in AVL) was tested, and for 50 also an oral, rectal and urogenital swab sample. For 32 animals only a blood sample was tested, and for 2 only oral, rectal and urogenital swab samples. For a subset of 68 animals, also a nested RT-PCR targeting the VP35 gene, as described in De Nys et al., 2019 (*4*), was carried out on RNA extracts from lung, spleen or DBS. Ninety-one fecal and one urine sample collected at the *E. helvum* colony were tested with both the abovementioned multiplex L gene qPCR as well as with the altona RealStar Filovirus Screen RT-PCR Kit 1.0 (altona Diagnostics, Hamburg, Germany), which contains an internal control. As this internal control did not amplify for 47 samples, we only consider the results of the 45 other samples that did not show signs of PCR inhibition. No RNA could be extracted from 48 sampled animals and three fecal samples due to either loss of the specimens or too little specimen available. In total, when counting each environmentally collected fecal sample as derived from a single, distinct individual, we tested samples of 465 different individuals by L gene qRT-PCR.

All extractions and qRT-PCR tests on animal tissue samples were performed at the Institut National de Recherche (INRB) in Kinshasa, DRC. All fecel samples were extracted at the Institut de Recherche pour le Développement (IRD), Montpellier, France and qRT-PCR-tested at the Institute for Tropical Medicine, Antwerp, Belgium.

Serology

272 DBS, 91 feces and one urine sample were tested for presence of antibodies against ebolaviruses using a Luminex-based assay as described in Ayouba et al., 2017 (*5*) and De Nys et al., 2019 (*4*). The assay is based on reaction with recombinant proteins NP, GP and VP40 of EBOV (Mayinga strain) and Sudan Ebola virus (SUDV), GP and VP40 of Bundibugyo virus (BDBV). Also GP of Reston virus (RESTV) and of EBOV Kissidougou strain were included (see Appendix Figure). For each run of the assay, we included one blank negative control (water), two sample negative controls (from a sample collected from bats hosted in a zoo in Montpellier, born in captivity there) and two positive controls (according to our positivity criteria) from bat field samples (see De Nys et al., 2019 (*4*)). Cutoff positivity values for the median fluorescence intensity (MFI) were determined as in De Nys et al., 2019 (*4*). All serology

tests were performed at the Institut de Recherche pour le Développement (IRD), Montpellier, France.

Molecular species identification

Cytochrome b (cytb) PCR was attempted on a subset of samples using primers cytb462F (5′-CGAAGCTTGATATGAAAAACCATCGTTG-3′) and cytb464R (5′-AGTGGRTTRGCTGGTGTRTARTTGTC-3′) on nucleic acid extractions from one of the extracted organ tissues and/or extracted DBS. If the cytb PCR failed, PCR with primers targeting the 16S gene (16S701F: 5′-CGGTTGGGGTGACCTCGGA-3′, 16S787R: 5′-AGATAGAAACCGACCTGGAT-3′) or the 12S gene (12S-L01091: 5′-AAAAAGCTTCAAACTGGGATTAGATACCCCACTAT-3′, and 12S-H01478: 5′-TGACTGCAGAGGGTGACGGGCGGTGTGT-3′) were attempted. These species identification PCRs and sequencing were conducted at IRD, Montpellier, France, and the Robert Koch Institute, Berlin, Germany.

All mitochondrial sequences were analyzed with NCBI BLAST (https://blast.ncbi.nlm.nih.gov/Blast.cgi) and aligned in Geneious v10.2 (https://www.geneious.com) together with representative sequences for the same or similar species downloaded from GenBank or the African Mammalia database (*6*) (http://projects.biodiversity.be/africanmammalia). Species assignment was based on percentage nucleotide sequence similarity and phylogenetic clustering following the latest genetic based taxonomic revisions in African mammal taxonomy (*7–17*). These sequences were then deposited under GenBank accession nos. MN597466–MN597893.

Maps

Maps in Figure 1 (main text) were made in R with ggmap (*18*) and rgdal (*19*) using base layers from OpenStreetMap (*20*) and Google.

Results and Discussion

Host Diversity and Frequency of Misidentifications in the Field

Out of 468 sampled individuals, for 334 we determined a mitochondrial genotype by sequencing part of the cytochrome b, 12S or 16S gene (Appendix Table). For most of these a species name or at least a temporary species reference name could be assigned, but for 4 species

no similar sequences at the species level were available in databases and hence were denoted by their genus and “sp1” (Appendix Table). For a further 25 non-genotyped animals the species could be unambiguously determined based on morphology, and for another 67 non-genotyped animals the genus could be unambiguously determined based on morphology. For one morphologically but not molecularly identified genus, *Graphiurus*, we only collected a single specimen, therefore inferring it represents a distinct species in our dataset. For 62 animals, no genus or species was determined.

In total we identified 47 different species from 34 different genera (Appendix Table). Only 91 individuals (84 bats, 2 rodents, 2 monkeys, 2 shrews, and the red river hog skull) were or could be correctly determined in the field to the species level based on morphology only (Appendix Table). Identification to the genus level based on morphology was possible, or done correctly, for 382 individuals (Appendix Table). For rodents, most genus misidentifications happened in the genera *Hybomys* (6/20), *Hylomyscus* (5/12) and *Praomys* (11/77) (Appendix Table). For bats, the most often misidentified genera were *Myotis*, misidentified in the field 3/3 times, and *Myonycteris* genus, misidentified 7/27 times. We therefore emphasize that infection surveillance in small mammals requires additional molecular-based identification of the host species.

Calculation of Sample Size Required to Detect Particular Prevalence Levels

To be able to detect at least one positive animal of a particular species with 95% level confidence, we could use the following equation to determine the necessary sample size N per assumed actual prevalence in the real reservoir species:

$$1 - (1 - p * s)^N > 0.95$$

with $p =$ prevalence, $s =$ test sensitivity and $N =$ number of tested individuals.

If EBOV would have circulated freely among all mammal species occurring at the time in the study area, our total negative sample of 465 RT-PCR tested specimens suggests a prevalence of at most 0.67%. However, we can assume EBOV transmission is in reality restricted to a limited number of host species only, therefore true prevalences could possibly have been higher while still being missed in our sample. Assuming a viral RNA or antibody prevalence (p) of e.g. 5% in the real reservoir species, and a test sensitivity of 95% (s), one would need a sample size N = 62 to be able to detect at least one positive animal with 95% confidence, and a sample size

of 314 when assuming a true prevalence of 1%. These sample sizes for these realistic prevalence levels were not reached for any species (Figure 2, main text; Appendix Table). For three species moderately good sample sizes were reached. For *Praomys jacksoni* (sensu stricto) and *Mus minutoides cf. gratus*, both rodents of the Muridae family, sample sizes were N = 53 and N = 54, so that we would have been able to detect at least one positive animal in each species with 95% confidence if the true prevalence was at least 5.7% (assuming s = 0.95), meaning that the true prevalences in these species at the study sites were lower than 5.7% and potentially zero. When combining *E. helvum* environmental feces (N = 45 successfully tested for EBOV RNA, assuming one fecal/urine sample per individual) and organ/blood tested *E. helvum* individuals (N = 14) a total sample size of that species of 59 was reached, from which we would have been able to detect at least one positive animal with 95% confidence if the true prevalence were 5.2%.

Discussion on the Animal Source of EBOV Infection of the Primary Case-Patient in the 2017 Likati Outbreak

The primary case-patient of the EBOV disease outbreak in Likati in April 2017 reportedly had been exposed to both the prepared meat of a hunted large bat, likely *E. helvum*, and a red river hog near his home village Kaigbono (see main text). The red river hog had been found dead and partly decomposed in the forest near Kaigbono village; and we indeed retrieved a red river hog skull at the described site at ≈700 m from the village. Reportedly, the meat at the side of the body touching the ground and the internal organs had been decomposed already, so only the upward shoulder had been taken for consumption. Up to four persons, none of whom fell ill, had carried out the butchering of suitable meat. The co-occurrence of an EBOV outbreak in humans around the same time makes it an acceptable possibility that this red river hog had died of EBOV disease. However, live EBOV might not have been present at the time of butchering: the described levels of decomposition could be expected in mammal carcasses in the tropics at ≈2–4 days post mortem (*21*,*22*); and in experimental conditions modeling temperature and humidity observed in West and Central Africa, EBOV survives <4 days in muscle tissue, though 7 days in blood (*23*). Neither swabs of the skull or the remains of the pulp of the molars tested positive for EBOV RNA, but it is possible that viral genomic RNA had degraded beyond detection level during the ≈4 months before sampling. Two of the people who butchered the hog's carcass prepared and cooked the meat, which subsequently was shared by ≈10–20 people of the village, including the primary case-patient (the only one to develop febrile illness).

The fact that the consumed hog meat had been cooked, that 10–20 people who were exposed to the same meat did not fall ill, and that the muscle had possibly been too old to contain viable EBOV, argue against the hog meat being the source of the infection. The bat on the other hand had been carried by the primary case-patient from the place at the Likati river where he received it. The bat had been hunted and killed by other persons, and its carcass prepared by his wife (internal organs removed and grilled), none of whom had reportedly fallen ill with febrile symptoms. The primary case was the only one to consume the grilled bat.

The ≈7 day time lag between consumption of the bat and onset of symptoms is more consistent with average EBOV incubation time, though also the ≈13 day time lag with the hog contact falls within range (*24*). The consumed bat likely belonged to the species *E. helvum*, as it was captured at or near an *E. helvum* colony site, with its high colony numbers and thus the relative ease in hunting these animals at the time, though other large bat species cannot be excluded. Our observations in July 2017 reveal that occasionally other species such as *Hypsignathus monstrosus* and *Epomops franqueti*, species previously found EBOV RNA positive (*25*), may have been hunted as well, but in much smaller numbers than *E. helvum*. *E. helvum* harbors an amino-acid change in its NPC1 protein (the main EBOV cell entry receptor) which greatly reduces the ability of EBOV to enter cells in culture compared to other tested bats species (*26*), and the GP gene sequence of the strain circulating in the 2017 Likati outbreak suggests this strain would also be refractory in *E. helvum* cells (*27*,*28*). However, anti-EBOV antibodies have been found in wild-caught *E. helvum* (*4*,*29*,*30*), so it seems EBOV or a closely related ebolavirus species is still able to infect this particular species in nature.

As none of the other individuals who had been in contact with the hog carcass, the hog's prepared meat, or the bat carcass reportedly had fallen ill, perhaps another unknown animal had infected the primary case of this Likati outbreak. However, we also do not know the serologic status of these exposed individuals. Neutralizing antibodies against EBOV could persist for many years post-infection (*31*), and antibodies against EBOV or other cross-reactive ebolavirus species are found in human populations without reported EBOV outbreaks (*32*). Immunological protection through prior EBOV exposure in some Kaigbono residents could explain why people in contact with the red river hog's or the fruit bat's unprepared carcasses did not develop symptoms. This could also explain why there was no transmission from the primary case-patient

to other Kaigbono residents, even the initial caretakers at his home, during his initial ≈7 days of illness spent in Kaigbono. Further transmission only occurred later outside the village to people transporting him on a motorbike to the hospital, although the patient's viral load must have been higher at this later stage.

In conclusion, despite the clear exposure of the primary case of the 2017 Likati outbreak to a red river hog and a fruit bat, likely *E. helvum*, neither the hog or the bat can be excluded nor confirmed as the source of the infection for the primary case, or if he had contracted EBOV from another animal during his routine activities.

References

1. Marí Saéz A, Weiss S, Nowak K, Lapeyre V, Zimmermann F, Düx A, et al. Investigating the zoonotic origin of the West African Ebola epidemic. EMBO Mol Med. 2015;7:17–23. PubMed https://doi.org/10.15252/emmm.201404792

2. Cnops L, Van den Eede P, Pettitt J, Heyndrickx L, De Smet B, Coppens S, et al. Development, evaluation, and integration of a quantitative reverse-transcription polymerase chain reaction diagnostic test for Ebola virus on a molecular diagnostics platform. J Infect Dis. 2016;214(suppl 3):S192–202. PubMed https://doi.org/10.1093/infdis/jiw150

3. de Wit E, Rosenke K, Fischer RJ, Marzi A, Prescott J, Bushmaker T, et al. Ebola laboratory response at the Eternal Love Winning Africa campus, Monrovia, Liberia, 2014–2015. J Infect Dis. 2016;214(suppl 3):S169–76. PubMed https://doi.org/10.1093/infdis/jiw216

4. De Nys HM, Kingebeni PM, Keita AK, Butel C, Thaurignac G, Villabona-Arenas CJ, et al. Survey of Ebola viruses in frugivorous and insectivorous bats in Guinea, Cameroon, and the Democratic Republic of the Congo, 2015–2017. Emerg Infect Dis. 2018;24:2228–40. PubMed https://doi.org/10.3201/eid2412.180740

5. Ayouba A, Touré A, Butel C, Keita AK, Binetruy F, Sow MS, et al. Development of a sensitive and specific serological assay based on Luminex technology for detection of antibodies to Zaire Ebola virus. J Clin Microbiol. 2016;55:165–76. PubMed https://doi.org/10.1128/JCM.01979-16

6. African Mammalia [cited 2019 Jun 13]. http://projects.biodiversity.be/africanmammalia

7. Bryja J, Mikula O, Šumbera R, Meheretu Y, Aghová T, Lavrenchenko LA, et al. Pan-African phylogeny of *Mus* (subgenus *Nannomys*) reveals one of the most successful mammal radiations in Africa. BMC Evol Biol. 2014;14:256. PubMed https://doi.org/10.1186/s12862-014-0256-2

8. Bryja J, Šumbera R, Kerbis Peterhans JC, Aghová T, Bryjová A, Mikula O, et al. Evolutionary history of the thicket rats (genus *Grammomys*) mirrors the evolution of African forests since late Miocene. J Biogeogr. 2017;44:182–94. https://doi.org/10.1111/jbi.12890

9. Mizerovská D, Nicolas V, Demos TC, Akaibe D, Colyn M, Denys C, et al. Genetic variation of the most abundant forest-dwelling rodents in Central Africa (*Praomys jacksoni* complex): evidence for Pleistocene refugia in both montane and lowland forests. J Biogeogr. 2019;46:1466–78. https://doi.org/10.1111/jbi.13604

10. Nicolas V, Schaeffer B, Missoup AD, Kennis J, Colyn M, Denys C, et al. Assessment of three mitochondrial genes (16S, Cytb, CO1) for identifying species in the Praomyini tribe (Rodentia: Muridae). PLoS One. 2012;7:e36586. PubMed https://doi.org/10.1371/journal.pone.0036586

11. Sabuni C, Aghová T, Bryjová A, Šumbera R, Bryja J. Biogeographic implications of small mammals from Northern Highlands in Tanzania with first data from the volcanic Mount Kitumbeine. Mammalia. 2018;82:360–72. https://doi.org/10.1515/mammalia-2017-0069

12. Missoup AD, Yemchui GD, Denys C, Nicolas V. Molecular phylogenetic analyses indicate paraphyly of the genus *Hybomys* (Rodentia: Muridae): taxonomic implications. J Zoological Syst Evol Res. 2018;56:444–52. https://doi.org/10.1111/jzs.12213

13. Stanley WT, Robbins LW, Malekani JM, Mbalitini SG, Migurimu DA, Mukinzi JC, et al. A new hero emerges: another exceptional mammalian spine and its potential adaptive significance. Biol Lett. 2013;9:20130486. PubMed https://doi.org/10.1098/rsbl.2013.0486

14. Stanley WT, Hutterer R, Giarla TC, Esselstyn JA. Phylogeny, phylogeography and geographical variation in the *Crocidura monax* (Soricidae) species complex from the montane islands of Tanzania, with descriptions of three new species. Zoological Journal of the Linnean Society. 2015;174:185–215. https://doi.org/10.1111/zoj.12230

15. Jacquet F, Denys C, Verheyen E, Bryja J, Hutterer R, Kerbis Peterhans JC, et al. Phylogeography and evolutionary history of the *Crocidura olivieri* complex (Mammalia, Soricomorpha): from a forest origin to broad ecological expansion across Africa. [PubMed]. BMC Evol Biol. 2015;15:71. PubMed https://doi.org/10.1186/s12862-015-0344-y

16. Hutterer R, Decher J, Monadjem A, Astrin J. A new genus and species of vesper bat from West Africa, with notes on *Hypsugo*, *Neoromicia*, and *Pipistrellus* (Chiroptera: Vespertilionidae). Acta Chiropterologica, 2019;21:1–22. https://doi.org/10.3161/15081109ACC2019.21.1.001

17. Patterson BD, Webala PW, Lavery TH, Agwanda BR, Goodman SM, Peterhans JCK, et al. Evolutionary relationships and population genetics of the Afrotropical leaf-nosed bats (Chiroptera, Hipposideridae). ZooKeys. 2020;929:117–61. PubMed https://doi.org/10.3897/zookeys.929.50240

18. Kahle D, Wickham H. Ggmap: spatial visualization with ggplot2. R J. 2013;5:144–61. https://doi.org/10.32614/RJ-2013-014

19. Bivand R, Keitt T, Rowlingson B, Pebesma E, Sumner M, Hijmans R, et al. Package 'rgdal'. bindings for the Geospatial Data Abstraction Library [cited 2017 Oct 15]. https://cran r-project org/web/packages/rgdal/index html

20. Open Street Map. [cited 2017 Aug 17] https://www.openstreetmap.org.

21. Amendt J, Zehner R, Krettek R. Insekten auf Leichen: Forensische Entomologie. Biologie in Unserer Zeit. 2005;35:232–40. https://doi.org/10.1002/biuz.200410284

22. Jirón LF, Cartín VM. Insect succession in the decomposition of a mammal in Costa Rica. Journal of the New York Entomological Society. 1981;89:158–65.

23. Prescott J, Bushmaker T, Fischer R, Miazgowicz K, Judson S, Munster VJ. Postmortem stability of Ebola virus. Emerg Infect Dis. 2015;21:856–9. PubMed https://doi.org/10.3201/eid2105.150041

24. Velásquez GE, Aibana O, Ling EJ, Diakite I, Mooring EQ, Murray MB. Time from infection to disease and infectiousness for Ebola virus disease, a systematic review. Clin Infect Dis. 2015;61:1135–40. PubMed https://doi.org/10.1093/cid/civ531

25. Leroy EM, Kumulungui B, Pourrut X, Rouquet P, Hassanin A, Yaba P, et al. Fruit bats as reservoirs of Ebola virus. Nature. 2005;438:575–6. PubMed https://doi.org/10.1038/438575a

26. Ng M, Ndungo E, Kaczmarek ME, Herbert AS, Binger T, Kuehne AI, et al. Filovirus receptor NPC1 contributes to species-specific patterns of ebolavirus susceptibility in bats. eLife. 2015;4:4. PubMed https://doi.org/10.7554/eLife.11785

27. Nsio J, Kapetshi J, Makiala S, Raymond F, Tshapenda G, Boucher N, et al. 2017 Outbreak of Ebola virus disease in northern Democratic Republic of Congo. J Infect Dis. 2020;221(5):701-6. PubMed

28. Wawina-Bokalanga T, Vanmechelen B, Martí-Carreras J, Vergote V, Vermeire K, Muyembe-Tamfum JJ, et al. Complete genome sequence of a new Ebola virus strain isolated during the 2017 Likati outbreak in the Democratic Republic of the Congo. Microbiol Resour Announc. 2019;8:e00360–19. PubMed https://doi.org/10.1128/MRA.00360-19

29. Ogawa H, Miyamoto H, Nakayama E, Yoshida R, Nakamura I, Sawa H, et al. Seroepidemiological prevalence of multiple species of filoviruses in fruit bats (*Eidolon helvum*) migrating in Africa. J Infect Dis. 2015;212(Suppl 2):S101–8. PubMed https://doi.org/10.1093/infdis/jiv063

30. Hayman DT, Emmerich P, Yu M, Wang LF, Suu-Ire R, Fooks AR, et al. Long-term survival of an urban fruit bat seropositive for Ebola and Lagos bat viruses. PLoS One. 2010;5:e11978. PubMed https://doi.org/10.1371/journal.pone.0011978

31. Rimoin AW, Lu K, Bramble MS, Steffen I, Doshi RH, Hoff NA, et al. Ebola virus neutralizing antibodies detectable in survivors of the Yambuku, Zaire outbreak 40 years after infection. J Infect Dis. 2018;217:223–31. PubMed https://doi.org/10.1093/infdis/jix584

32. Mulangu S, Alfonso VH, Hoff NA, Doshi RH, Mulembakani P, Kisalu NK, et al. Serologic evidence of Ebolavirus infection in a population with no history of outbreaks in the Democratic Republic of the Congo. J Infect Dis. 2018;217:529–37. PubMed https://doi.org/10.1093/infdis/jix619

Appendix Table. Overview of mammalian species tested for EBOV and SUDV, Likati Health Zone, 2017*

Order	Taxon	Species correctly identified in field | not identified in field, no. specimens	Genus correctly identified in field | not identified in field, no. specimens	Genotyped mitochondrial gene, no. specimens	Tested for EBOV and SUDV RNA by qRT-PCR, no. specimens	Tested with Luminex for antibodies against EBOV, SUDV, BDBV, RESTV, no. specimens
Artiodactyla	*Potamochoerus porcus*	1 | 0	1 | 0	0	1	0
Carnivora	*Atilax paludinosus*	0 | 1	0 | 1	1	1	1
Chiroptera	*Casinycteris argynnis*	5 | 6	9 | 2	10	10	11
	Chiroptera (family/genus/species ND)	-	-	-	2	2
	Doryrhina cyclops	10 | 2	11 | 1	12	11	12
	Eidolon helvum	12 | 2	14 | 0	11	14	12
	Environmental faeces *E. helvum* colony	0 | 0	0 | 0	0	45	92
	Epomops franqueti	31 | 0	31 | 0	27	22	25
	Glauconycteris egeria	0 | 1	0 | 1	1	1	1
	Hipposideridae (genus/species ND)	-	-	-	4	2
	Hipposideros beatus	0 | 1	1 | 0	1	1	0
	Hipposideros fuliginosus	0 | 1	1 | 0	1	1	1
	Hypsignathus monstrosus	5 | 0	5 | 0	4	5	5
	Macronycteris gigas	7 | 0	7 | 0	5	5	7
	Megaloglossus woermanni	9 | 2	11 | 0	7	10	11
	Myonycteris torquata	4 | 23	20 | 7	20	17	21
	Myotis bocagii	0 | 3	0 | 3	3	3	2
	Neoromicia nanus	0 | 1	0 | 1	1	1	1
	Parahypsugo sp.1	0 | 1	0 | 1	1	1	1
	Pteropodidae (genus/species ND)	-	-	-	1	1
	Rhinolophidae (genus/species ND)	-	-	-	1	2
	Rhinolophus alcyone	1 | 2	3 | 0	3	3	3
	Rhinolophus sp.1	0 | 1	0 | 1	1	1	1
	Vespertilionidae (genus/species ND)	0 | 0	0 | 0	0	1	0
Eulipotyphla	*Crocidura* (species ND)	-	42 | 0	0	34	25
	Crocidura caliginea	0 | 8	8 | 0	8	8	6
	Crocidura crenata	0 | 3	3 | 0	3	3	1
	Crocidura denti	2 | 4	6 | 0	6	6	0
	Crocidura latona sensu Stanley et al. 2015	0 | 2	2 | 0	2	2	0
	Crocidura latona sensu Willows-Munro et al. 2011	0 | 1	1 | 0	1	1	0
	Crocidura ludia	0 | 2	2 | 0	2	2	0
	Crocidura olivieri clade IV sensu Jacquet et al 2015	0 | 10	10 | 0	10	10	9
	Paracrocidura schoutedeni	0 | 1	1 | 0	1	1	1
	Paracrocidura sp.1	0 | 1	0 | 1	1	1	0
	Scutisorex somereni	0 | 3	3 | 0	3	3	2
Primates	Cercopithecus (species ND)	-	2 | 0	0	0	0
	Cercopithecus ascanius	2 | 0	2 | 0	2	2	0
	Colobus (species ND)	-	1 | 0	0	0	0
Rodentia	*Anomalurus derbianus*	1 | 0	1 | 0	0	1	0
	Atherurus africanus	0 | 1	1 | 0	0	1	0
	Funisciurus sp.1	0 | 2	2 | 0	2	2	1
	Grammomys p2 sensu Bryja et al. 2017	0 | 2	1 | 1	2	2	1
	Graphiurus sp.1	0 | 1	1 | 0	0	1	1

Order	Taxon	Species correctly identified in field | not identified in field, no. specimens	Genus correctly identified in field | not identified in field, no. specimens	Genotyped mitochondrial gene, no. specimens	Tested for EBOV and SUDV RNA by qRT-PCR, no. specimens	Tested with Luminex for antibodies against EBOV, SUDV, BDBV, RESTV, no. specimens
	Hybomys lunaris	0 | 20	14 | 6	20	20	5
	Hylomyscus alleni	0 | 5	3 | 2	5	5	1
	Hylomyscus stella	0 | 7	4 | 3	7	7	2
	Lophuromys (species ND)	-	3 | 0	0	2	2
	Lophuromys dudui	1 | 13	14 | 0	14	14	6
	Malacomys (species ND)	0 | 0	1 | 0	0	1	1
	Malacomys longipes	0 | 1	0 | 1	1	1	1
	Mastomys natalensis	0 | 2	1 | 1	2	2	0
	Muridae (genus/species ND)	-	-	-	27	28
	Mus (*Nannomys*) (species ND)	0 | 0	17 | 0	0	11	9
	Mus (*Nannomys*) *bufo*	0 | 1	1 | 0	1	1	0
	Mus (Nannomys) minutoides cf. gratus	0 | 54	54 | 0	54	54	19
	*Praomys jackson*i sensu lato clade IVc	0 | 14	14 | 0	14	14	3
	Praomys jacksoni sensu stricto clade Ib	0 | 53	44 | 9	53	53	20
	Praomys misonnei	0 | 9	7 | 2	9	9	5
	Praomys verschureni	0 | 1	1 | 0	1	1	0
	Stochomys longicaudatus	0 | 1	1 | 0	1	1	1
Total		91 | 269	382 | 44	334	465	364

*Overview of sampled mammal species, or the lowest taxonomic rank to which a specimen (i.e. an individual animal) could be assigned, numbers of specimens for which identification to species or genus level was possible or performed correctly in the field versus when identification was not possible or done correctly in the field (numbers separated by "|"), number of specimens per taxon for which the species was determined or confirmed via a sequence of the cytochrome b, 12S or 16S mitochondrial genes, and number of specimens tested for EBOV and SUDV by qRT-PCR and for anti-ebolavirus antibodies by Luminex assay. See Appendix for a discussion on species identifications in the field versus via mitochondrial genotype. BDBV, Bundibugyo virus; EBOV, Ebola virus; ND, Not determined; qRT-PCR, quantitative reverse transcription PCR; RESTV, Reston virus; SUDV, Sudan virus. Dashes indicate cells without applicable data.

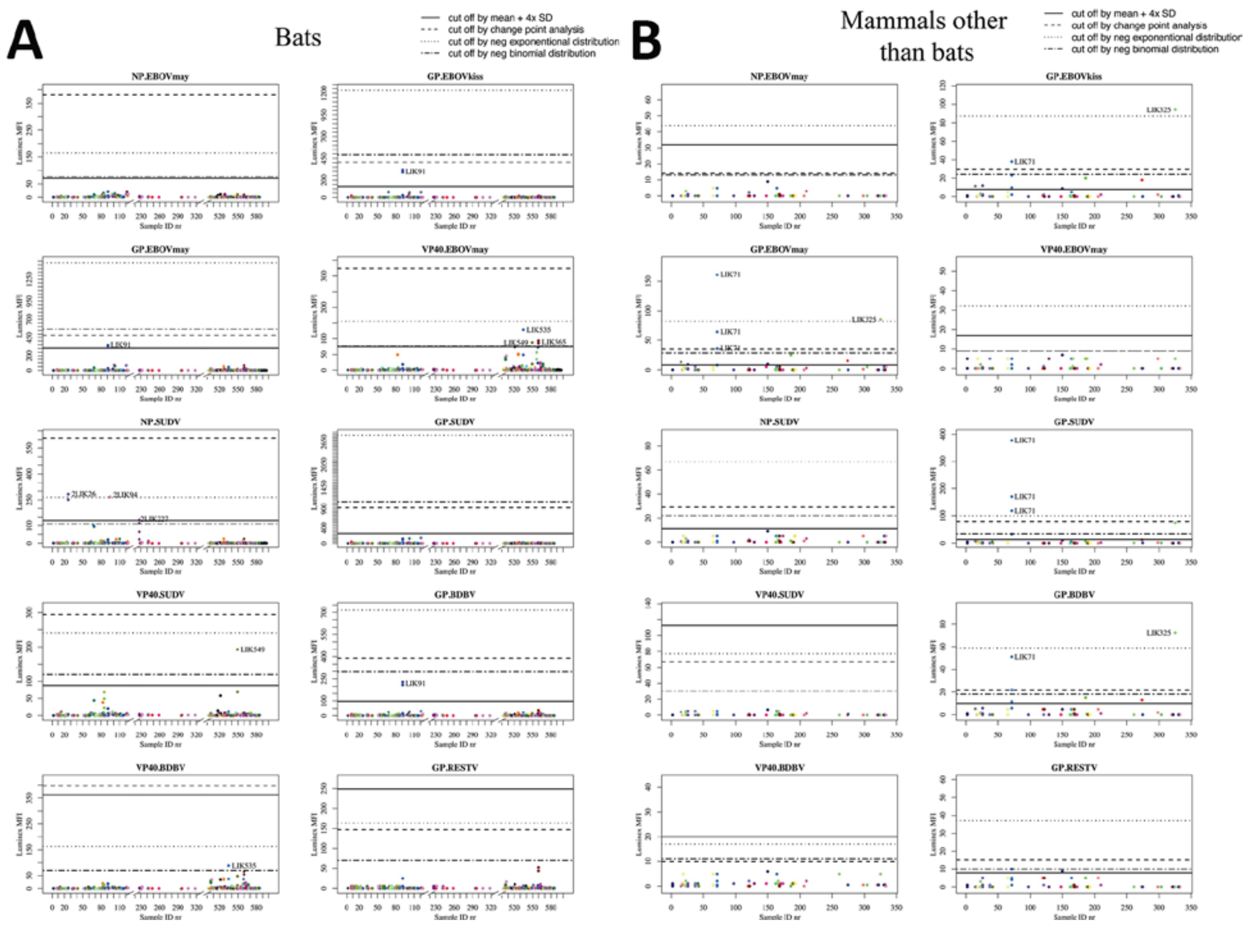

Appendix Figure. Median fluorescent intensity (MFI) of dried blood spot and fecal samples interacting with ten different antigens in a Luminex assay (*4*) for bats (A) and for other mammal samples (B). MFI values are presented for each antigen in a separate plot. Antigens are recombinant proteins: the nucleoprotein of Ebola virus (EBOV) Mayinga strain (NP.EBOVmay), Sudan virus (SUDV) (NP.SUDV); glycoprotein of EBOV Mayinga strain (GP.EBOVmay), EBOV Kissidougou-Makona strain (GP.EBOVkiss), SUDV (GP.SUDV), Bundibugyo virus (GP.BDBV), and Reston virus (GP.RESTV); and viral matrix protein of EBOV Mayinga strain (VP40.EBOVmay), SUDV (VP40.SUDV), and Bundibugyo virus (VP40.BDBV). Cutoff values were determined separately for samples of bats and samples of other mammals, mostly rodents and shrews. Each individual animal has its own arbitrary color code and is arranged according its ID number along the x-axis. Multiple samples tested from the same individual are thus arranged vertically. Although blood samples of 4 bats, 1 mongoose, and 1 shrew showed MFI levels above the calculated cutoff levels for at ≥1 tested antigen and 1 test (tests for these samples were repeated 2–4 times when enough material was available), we remain cautious to denote these samples as testing positive for antibodies against an ebolavirus. These samples do not fulfill the Luminex assay positivity criteria defined in earlier works (*3*,*4*) because they only reacted against 1 of 3 antigens per EBOV lineage from which these proteins are derived. ID, identification.
